# A mini-review on mobile manipulators with Variable Autonomy

**DOI:** 10.3389/frobt.2025.1540476

**Published:** 2025-02-14

**Authors:** Cesar Alan Contreras, Alireza Rastegarpanah, Manolis Chiou, Rustam Stolkin

**Affiliations:** ^1^ School of Metallurgy and Materials, The University of Birmingham, Birmingham, United Kingdom; ^2^ School of Computing and Digital Technology, Birmingham City University, Birmingham, United Kingdom; ^3^ School of Electronic Engineering and Computer Science, Queen Mary University of London, London, United Kingdom

**Keywords:** mobile manipulators, uncertain environments, variable autonomy, human-robot teaming, shared control, human-robot interaction

## Abstract

This paper presents a mini-review of the current state of research in mobile manipulators with variable levels of autonomy, emphasizing their associated challenges and application environments. The need for mobile manipulators in different environments, especially hazardous ones such as decommissioning and search and rescue, is evident due to the unique challenges and risks each presents. Many systems deployed in these environments are not fully autonomous, requiring human-robot teaming to ensure safe and reliable operations under uncertainties. Through this analysis, we identify gaps and challenges in the literature on Variable Autonomy, including cognitive workload and communication delays, and propose future directions, including whole-body Variable Autonomy for mobile manipulators, virtual reality frameworks, and large language models to reduce operators’ complexity and cognitive load in some challenging and uncertain scenarios.

## 1 Introduction

Robots are deployed in different environments to aid and complement humans in tasks, including manufacturing (Rajendran et al., 2021), healthcare (Lin et al., 2020), and agriculture (Chen et al., 2022), where the benefits of automation are observable. Robots have also been used in more challenging scenarios. For example, mobile robots and mobile manipulators deployed in disaster zones (Chen and Cho, 2019) or in other extreme environments such as nuclear disaster response or decommissioning (Nagatani et al., 2013; Chiou et al., 2022; Cragg and Hu, 2003) excel because of their mobility and manipulation capabilities. However, despite their potential, uncertainties prevent these systems from being fully autonomous. Human intervention remains essential due to a lack of trust and the limitations of current autonomous systems.

The deployment of autonomous robots across environments shows that no single solution fits all needs. Autonomous systems are limited by their prior knowledge and adaptive capabilities, with training being difficult and time-consuming. Learning from demonstration approaches (Moridian et al., 2018) are often confined to their training environments and require human intervention for decisions beyond their training. Improvements in path planning for mobile bases and manipulators (Rajendran et al., 2021; Hargas et al., 2015; Chen et al., 2022) also face limitations needing human intelligence. Manual operation requires significant training and concentration, with cognitive demands varying between operators. These cognitive challenges make manual systems more error-prone than semi-autonomous or fully autonomous systems (Rastegarpanah et al., 2024; Chiou et al., 2015).

Robots deployed in complex environments increase operator demands for alertness (Chiou, 2017), adaptability to new information (Rastegarpanah et al., 2024), and concentration due to unexpected delays and drops in connection (Cragg and Hu, 2003). These burdens can cause physical fatigue and monotony. Some challenges can be alleviated by balancing teleoperation and autonomy, as proposed by field exercises (Chiou et al., 2022), and reinforced by our anecdotal interactions with operators in Japan preparing for a teleoperated deployment at Fukushima Daiichi Nuclear Power Plant. The operators noted that these three factors are needed due to environmental uncertainties and issues with repetitiveness and communication delays. Automating simple, repetitive processes can address some issues, while other conditions require manual control. Given the challenges for fully autonomous and manual systems, developing systems that can switch between human control and autonomy is logical. Mobile bases give the freedom to explore environments, while manipulators enable interaction with objects within them, making mobile manipulators a system worth studying within this context.

Previous reviews on Variable Autonomy (VA) have focused on cognitive aspects, methodologies, and applications but not specifically on mobile manipulators. For instance, Tabrez et al. (2020) examined mental models and their traits like fluency, adaptability, and effective communication. Villani et al. (2018) discuss cognitive and physical aspects of programming collaborative and shared control robots in industrial settings, emphasizing safe interaction and intuitive interfaces. Bengtson et al. (2020) focused on computer vision for semi-autonomous control of assistive robots, while Moniruzzaman et al. (2022) focus on the teleoperation of mobile robots. On the other hand, reviews on mobile manipulators have focused on motion planning (Sandakalum and Ang, 2022) and the decision-making process of planning algorithms (Thakar et al., 2023) with limited coverage of human-robot interaction, and Variable Autonomy.

Our previous work has addressed varying levels of autonomy in disaster and rescue scenarios, focusing on cognitive and robotic challenges within this scope, limited to mobile robots (Chiou, 2017; Chiou et al., 2021; Panagopoulos et al., 2022; Ramesh et al., 2023). However, there is a need to expand this understanding to other environments where human-robot teams are deployed and mobile manipulators are used. Mobile manipulators can function as single-entity systems, where locomotion and manipulation are coupled, or as dual-entity systems, treating the base and manipulator separately. With this mini-review, we aim to 1) present the current state of research, 2) identify some challenges, insights, and gaps from the current literature, and 3) propose future research directions with a focus on mobile manipulators, their control within human-robot teams, and Variable Autonomy.

### 1.1 Methodology

For the review, we performed a Google Scholar search, with the specifics of our search criteria found in Table 1. We define mobile manipulators as robots with locomotion decoupled from manipulation, moving primarily on the ground. This definition includes humanoid robots, quadruped robots, wheeled manipulators, and robots on tracks. For the manipulation system, we consider any robot capable of performing tasks typically done by a hand, such as throwing, pushing, grasping, cutting, etc. The system does not necessarily need to perform any manipulation or mobile base task, but must be physically capable to do so.

**TABLE 1:** Search and Exclusion Criteria for the mini-review on Mobile Manipulators with Variable Autonomy.

Search criteria	
Search terms	Results
“Mobile manipulator” and “variable autonomy”	24
“Mobile manipulator” and “autonomy levels”	41
“Mobile manipulator” and “shared autonomy”	235
Period	January 2018 – June 2024
Search engines	Google scholar
Publication type	Peer-reviewed papers, academic papers, conference papers, journal articles, theses
Exclusion criteria	
Language	Non-English
Contextual	Studies involving robots which are not contained in the mobile manipulators (e.g., aerial vehicles, underwater robots, soft robots)
Scope	Excluded papers not addressing or utilizing advancements in Variable Autonomy (e.g., mixed initiative, shared control, etc.)

This definition excludes robots with integrated or inseparable locomotion and manipulation systems, such as snake, octopus-inspired, and soft robots. It also excludes aerial and underwater robots and stationary robotic arms without a mobile base.

Based on the number of search results, most research is centred around shared autonomy rather than mixed initiative or other forms of Variable Autonomy. After applying the exclusion and contextual criteria, 38 papers were included in the review.

## 2 Literature review

Variable Autonomy systems enable flexible control by humans and machines across different levels of operation. Although various definitions exist for Variable Autonomy in the literature (Reinmund et al., 2024; Methnani et al., 2024), we classify these systems into two primary categories. The first is autonomy-changing systems, where autonomy levels can be modified during task execution: Full VA or Mixed-Initiative systems allow humans and machines to adjust these levels. Only humans can make autonomy changes in human-initiative (HI) or adjustable autonomy systems, whereas AI-initiative (AI-I) or sliding autonomy systems permit machines to do them. The second category is autonomy-sharing systems, often called Shared Control systems. In these systems, humans and robots work together on tasks. Shared Control can be either supervisory, where humans provide high-level directives, or assistive, where humans directly control the robot with system support, such as visual feedback or trajectory guidance.

### 2.1 Environments

The first focus of this paper was on robotic deployment environments, analyzing works detailing their application methodologies. This condition identified seven primary environments: hazardous materials and environments handling, disaster response, industrial manufacturing, Research and Development (R&D) in laboratories, healthcare and medical applications, agriculture or farming, and domestic/household environments. Table 2 lists the papers by environment and briefly describes their applications.

**TABLE 2:** Summary of papers, environments, and key findings.

Papers	Environment	Summary
Frese et al. (2022), Roennau et al. (2022), Schuster et al. (2020), Wedler et al. (2021), Woock et al. (2022)	Hazardous materials handling	Discuss the use of mobile manipulators in handling hazardous materials, examples include digging contaminated soil or waste (Frese et al., 2022; Woock et al., 2022), grasping hazardous objects (Roennau et al., 2022), and taking space soil samples (Schuster et al., 2020; Wedler et al., 2021)
Chiou et al. (2022), Frering et al. (2022), Verhagen et al. (2024)	Disaster response	Applications examples include the deployment of mobile manipulators for exploration and mapping of destroyed nuclear facilities (Chiou et al., 2022) and scenarios for fire fighting (Frering et al., 2022; Verhagen et al., 2024)
Merkt et al. (2019), Sirintuna et al. (2024), Stibinger et al. (2021)	Industrial manufacturing	Examine applications in industrial manufacturing, examples include performing tasks on moving assembly components (Merkt et al., 2019), collaborative transportation of large objects (Sirintuna et al., 2024) and placement of construction materials (Stibinger et al., 2021)
Moridian et al. (2018), Hargas et al. (2015), Båberg (2022); Baek et al. (2022), Benzi et al. (2022), Chen et al. (2018), Cheong et al. (2021), Fozilov et al. (2021), Gholami et al. (2020), Li et al. (2024), Palan et al. (2019), Valner et al. (2018), Wong et al. (2022)	Research and development laboratories	Reviews the use of mobile manipulators in R&D labs, focusing on research and experiments not for a specific deployment. Examples include works on teleoperation (Båberg, 2022; Chen et al., 2018; Cheong et al., 2021; Gholami et al., 2020; Li et al., 2024; Valner et al., 2018), path planning (Moridian et al., 2018; Baek et al., 2022; Hargas et al., 2015; Palan et al., 2019), whole-body control (Benzi et al., 2022; Wong et al., 2022), and multi-robot coordination (Fozilov et al., 2021)
Lin et al. (2020), Abubakar et al. (2020), Kapusta and Kemp (2019), Sanchez and Smart, (2021), Sanchez and Smart, (2022)	Healthcare and medical areas	Investigates applications in healthcare, including nursing, patient care (Abubakar et al., 2020; Kapusta and Kemp, 2019; Lin et al., 2020) and surface disinfection (Sanchez and Smart, 2021; 2022)
Chen et al. (2022)	Agriculture and farming	Studies the use in agricultural settings, in this case, the example applies to the pruning of a tree (Chen et al., 2022)
Bhattacharjee et al. (2020), Karim et al. (2023), Kemp et al. (2022), Kim et al. (2023), Mirjalili et al. (2024), Park et al. (2020), Pohl et al. (2024), Rakita et al. (2019)	Domestic and household environments	Includes the use of mobile manipulators in the house, examples include feeding assistance (Bhattacharjee et al., 2020; Karim et al., 2023; Park et al., 2020), and general house grasping and transportation tasks (Kemp et al., 2022; Kim et al., 2023; Mirjalili et al., 2024; Pohl et al., 2024; Rakita et al., 2019)

### 2.2 Tasks that variable autonomy tackles

After categorizing the papers by environment, the next step is to categorize them by the tasks to which Variable Autonomy is applied. This categorization means focusing on how Variable Autonomy and human-robot interaction are utilized as tools to accomplish various tasks. In other words, while some papers include Variable Autonomy, they do so to aid in completing other tasks and not necessarily researching ways to change the autonomy levels.

#### 2.2.1 Human mapping movement

Humanoid robots often take inspiration from human capabilities (Lin et al., 2020; Baek et al., 2022; Pohl et al., 2024). Some of these systems use motion mapping based on human posture or control (Rastegarpanah et al., 2016). For instance, Baek et al. (2022) utilize human leaning to control velocity while avoiding obstacles. In this approach, the robot provides a force feedback based on proximity to obstacles, allowing users to adjust their input. The robot can also alter its own velocity and path. Similarly, Lin et al. (2020) map human movements to specific humanoid movements to simplify a pick-and-place task. In their system, humans only need to make physical movements and signs that show a decision, and robots autonomously complete the task.

#### 2.2.2 Manipulation

Research in manipulation includes grasping, autonomous manipulation, and load balancing. This type of research is characterized by robots performing tasks with some level of autonomy. Papers that involve manipulation research in any form include (Lin et al., 2020; Chen et al., 2022; Hargas et al., 2015; Frese et al., 2022; Roennau et al., 2022; Rastegarpanah et al., 2021; Schuster et al., 2020; Wedler et al., 2021; Merkt et al., 2019; Stibinger et al., 2021; Cheong et al., 2021; Fozilov et al., 2021; Kapusta and Kemp, 2019; Sanchez and Smart, 2021; 2022; Mirjalili et al., 2024; Park et al., 2020; Pohl et al., 2024; Rakita et al., 2019). Unlike teleoperation research, in this area, the robot completes most of the manipulation tasks by itself. For example, Frese et al. (2022) use a giant excavator that plans how to dig soil, understands its properties, and balances the load. The system allows it to ask for the help of an operator if there is a low probability of success. Another example is Cheong et al. (2021), who allows the operator to select the object he wants to manipulate, with the robot autonomously extracting key points and finding grasp candidates.

#### 2.2.3 Transportation

This area includes path planning that considers the geometry and physical properties of the objects being moved, as well as obstacles in the path of the robot or object (Woock et al., 2022; Sirintuna et al., 2024; Benzi et al., 2022; Abubakar et al., 2020). For example, ARNA, from Abubakar et al. (2020), can transport an object while assisting a patient walking through a scene. In this case, the patient only controls the direction, while the robot independently manipulates and transports the object. Sirintuna et al. (2024) proposed a collaborative approach where the robot provides haptic feedback through a belt worn by a human in an occluded environment. Assisting them in transporting an object collaboratively with the robot by feeling a force when obstacles get closer. The human commands the direction, while the robot provides environmental information and transports the vehicle with a fixed end-effector position relative to the base.

### 2.3 Challenges and techniques VA aids with

This subsection explores areas where VA has provided critical support, including collision avoidance, communication handling, semantic understanding, and intent recognition.

#### 2.3.1 Collision, obstacle avoidance, mapping and navigation

This area utilizes sensor integration and real-time processing to enable robots to make decisions and adjust their path to avoid collisions. Relevant papers include (Hargas et al., 2015; Frese et al., 2022; Roennau et al., 2022; Woock et al., 2022; Cheong et al., 2021; Fozilov et al., 2021; Gholami et al., 2020; Valner et al., 2018; Kapusta and Kemp, 2019). Roennau et al. (2022) describe a system where an operator selects an object to retrieve, and the robot plans the path using a 3D SLAM-generated map to avoid collisions. Another example is Valner et al., 2018 who developed a framework where the machine autonomously switches sensor feed to another if one fails. The human operator provides high-level commands, asking the robot to capture the environment while the system handles mapping.

#### 2.3.2 Communication and delays

The impact of communication delays is discussed by various researchers (Frese et al., 2022; Merkt et al., 2019; Båberg, 2022; Li et al., 2024; Valner et al., 2018). Båberg (2022) have shown some work in user interfaces that helps an operator assess network reliability. Frese et al. (2022) depend on hardware communication speeds with buffer configuration and pre-allocation of memory. Variable Autonomy can help mitigate delays by providing autonomous control when high latency is detected, running directly on the robot’s internal systems, while allowing long-distance manual control when latency is low.

#### 2.3.3 Semantics and machine learning

This area takes advantage of the computational power for object recognition (Lin et al., 2020; Woock et al., 2022; Cheong et al., 2021; Bhattacharjee et al., 2020; Park et al., 2020; Pohl et al., 2024), task learning (Wong et al., 2022; Park et al., 2020; Rakita et al., 2019), and the use of Large Language Models (LLMs) (Kim et al., 2023; Mirjalili et al., 2024) for developing smarter systems. The primary focus of this area is helping humans reduce their cognitive load; smarter systems can allow humans to take a supervisory role in tasks and only take full control when an object or task not previously trained for is encountered. As an example, Bhattacharjee et al. (2020) allow a user to select a food from an interface, limiting its choices to some fruits detected by a perception algorithm but allowing the user to take manual control of other feeding processes.

#### 2.3.4 Teleoperation modes, and intent recognition

Research areas that allow humans to manually control a robotic system from a distance or share control with the robot (Schuster et al., 2020; Verhagen et al., 2024; Baek et al., 2022; Chen et al., 2018; Gholami et al., 2020; Li et al., 2024; Wong et al., 2022; Bhattacharjee et al., 2020; Kemp et al., 2022). Some researchers focus on providing high-level commands, enabling the robot to execute pre-programmed tasks while they explore other methods to communicate their intentions. Bhattacharjee et al. (2020) employ voice commands, Wong et al. (2022) try influencing a robot with physical touch, and Chen et al. (2018) propose utilising hand gestures. Other researchers use computer assistance for specific tasks, while manually moving the robots. Li et al. (2024) manage teleoperation of the mobile base and manipulator arm independently but use the system to decide when to switch between devices.

## 3 Discussion, insights and challenges

The explored literature on Variable Autonomy for mobile manipulators is divided into two focuses: 1) high-level control, or supervisory control, and 2) low-level control or system assistance. Most implementations involving manipulation, obstacle avoidance, mapping, transportation, and machine learning research aim for fully automated tasks. In these cases, the role of the operator is primarily to decide, choose tasks, or supervise to ensure the robot is not making mistakes. For known problems, this solution is good, providing automated solutions that are easy to use. On the other hand, we have teleoperated scenarios, often with some uncertainty. In these, humans drive the base or move the arm, with autonomy serving in an assistive capacity, with the main objective of lowering human cognitive load or reducing the operation completion time.

Separate Focus on Base and Manipulator - In mobile manipulators, Variable Autonomy is still primarily focused on controlling the base or the manipulator separately. Current research does not consider the joint problem of integrating changes in autonomy for both. This can be seen in a multitude of papers including: (Lin et al., 2020; Frese et al., 2022; Woock et al., 2022; Merkt et al., 2019; Båberg, 2022; Cheong et al., 2021; Fozilov et al., 2021; Gholami et al., 2020; Palan et al., 2019; Valner et al., 2018; Sanchez and Smart, 2022; Bhattacharjee et al., 2020; Karim et al., 2023; Kemp et al., 2022; Kim et al., 2023; Mirjalili et al., 2024; Park et al., 2020; Pohl et al., 2024; Rakita et al., 2019). Researchers in this area focus on applying varying levels of autonomy to either of the systems while keeping the rest of the robot static or at the same autonomy level throughout the task. Even transportation tasks follow a sequential process of changing between both: reaching a position with the base, picking the object with the manipulator, reaching a dropping position with the base, and placing the object with the manipulator. This approach can theoretically limit the operational workspace of a mobile manipulator. For example, in Sanchez and Smart (2022), the disinfection area is limited because the mobile base is not used simultaneously to increase the reach of the manipulator.

Human Cognitive Load - Refers to the mental effort required to perform a task and is a term acknowledged and investigated in multiple papers including, (Chiou, 2017; Sirintuna et al., 2024; Baek et al., 2022; Lin et al., 2020). However, it is still primarily studied using subjective measurements, such as the NASA Task Load Index (NASA-TLX). Currently, objective metrics and biometric data from the human operator are not widely used in systems of mobile manipulators with variable autonomy or involving human-in-the-loop operations. Implementing objective data from the participants would enhance our understanding of cognitive load and help design better support for human operators.

Communication Delays and System Reliability - Although known to cause issues, they are often ignored or not measured in implementations of mobile manipulation. There is a lack of studies addressing this problem in relevant environments. Moniruzzaman et al. (2022) mention compensation techniques, such as future pose estimation and point-cloud 3D reconstruction, that could benefit the area if applied. In addition, Variable Autonomy could be used by switching from manual teleoperation to a local compensation algorithm when higher latency is detected.

Uncertain Environments - Applying varying levels of autonomy in known environments, where high-level and supervisory control is feasible, is a popular and researched area. The challenge lies in extending high-level control strategies to more complex and unpredictable environments, where robust decision-making and adaptability matter.

### 3.1 Future work

In future work, several key directions merit attention to advance the field further. First, better system integration is essential, with research focusing on enabling simultaneous control of both the base and manipulator, whether coupled or decoupled. Such integration would facilitate switching between different levels of autonomy for each component. Building on existing work that allows the autonomous switching of operator control between the base and manipulator (Li et al., 2024), this approach could expand the operational range to larger manipulation workspaces.

Second, Virtual Reality (VR) offers significant potential in this domain. Current studies already highlight VR’s role in reducing cognitive load and enhancing environmental awareness (Baek et al., 2022; Woock et al., 2022; Rastegarpanah et al., 2024). Future research could delve deeper into its application in more complex mobile manipulation problems. Incorporating considerations like world physics and virtual world design metrics could create a smoother and more intuitive operator experience.

Another promising avenue lies in employing machine learning to address delays and errors. Techniques like intent recognition, already used in task assignment and reward function learning (Gholami et al., 2020; Palan et al., 2019), could be further developed to manage tasks typically dropped due to latency. By integrating onboard autonomous compensation algorithms with manual teleoperation, long-distance systems could benefit from reduced errors and delays. Switching between autonomous and manual modes could offer additional resilience.

Finally, the increasing capability of Large Language Models (LLMs) presents an exciting opportunity. As these models evolve to become multimodal, they can serve as versatile general assistants. Research has shown their potential for contextual awareness (Kim et al., 2023), which could be leveraged to enhance task awareness and dynamically adjust autonomy levels based on previously unconsidered data. This adaptability could enable the development of on-demand algorithms, significantly improving the flexibility and efficiency of mobile manipulators.

### 3.2 Conclusion

This mini-review synthesized current research on mobile manipulators with Variable Autonomy, revealing gaps and possible opportunities. The gaps included: First, Variable Autonomy and control in mobile manipulators often focus separately on the base and the manipulator. However, some challenges, such as cutting in large surfaces (Pardi et al., 2020) and cleaning of contaminated areas (Sanchez and Smart, 2022), require control of both and changes in both simultaneously. Second, studies on cognitive workload are heavily based on subjective metrics. However, some tasks with operators in the loop in hazardous environments would heavily benefit from real-time objective metrics during robot deployment (Chiou et al., 2022), as this could aid in better setting the autonomy levels in the system affected by operator load. Third, communication delays and reliability issues are acknowledged but not extensively addressed, which can be a very important factor to consider in time-critical situations (Moniruzzaman et al., 2022) that search and rescue or hazardous environments can have. Finally, most of the research is designed for static and known environments, lacking implementations in uncertain environments, when most of the research in this area is needed for situations under heavy uncertainties (search and rescue, manufacturing, decommissioning) (Båberg, 2022; Rajendran et al., 2021; Woock et al., 2022). Future research should aim to develop integrated variable autonomy for both the base and the manipulator, use VR or other intuitive interfaces as a way to deal with workload and facilitate shared control on the robot systems, implement adaptive communication protocols or change autonomy levels in the robot to handle network instability and implement real-time general decision-making frameworks based on LLMs that dynamically adjust autonomy levels based on situational and contextual demands.
